# Combining a Spatial Model and Demand Forecasts to Map Future Surface Coal Mining in Appalachia

**DOI:** 10.1371/journal.pone.0128813

**Published:** 2015-06-19

**Authors:** Michael P. Strager, Jacquelyn M. Strager, Jeffrey S. Evans, Judy K. Dunscomb, Brad J. Kreps, Aaron E. Maxwell

**Affiliations:** 1 Division of Resource Management, West Virginia University, Morgantown, West Virginia, United States of America; 2 Natural Resource Analysis Center, West Virginia University, Morgantown, West Virginia, United States of America; 3 The Nature Conservancy, Fort Collins, Colorado, and Department of Zoology and Physiology, University of Wyoming, Laramie, Wyoming, United States of America; 4 The Nature Conservancy, Charlottesville, Virginia, United States of America; 5 Alderson Broaddus University, Philippi, West Virginia, United States of America; Universidade de Vigo, SPAIN

## Abstract

Predicting the locations of future surface coal mining in Appalachia is challenging for a number of reasons. Economic and regulatory factors impact the coal mining industry and forecasts of future coal production do not specifically predict changes in location of future coal production. With the potential environmental impacts from surface coal mining, prediction of the location of future activity would be valuable to decision makers. The goal of this study was to provide a method for predicting future surface coal mining extents under changing economic and regulatory forecasts through the year 2035. This was accomplished by integrating a spatial model with production demand forecasts to predict (1 km^2^) gridded cell size land cover change. Combining these two inputs was possible with a ratio which linked coal extraction quantities to a unit area extent. The result was a spatial distribution of probabilities allocated over forecasted demand for the Appalachian region including northern, central, southern, and eastern Illinois coal regions. The results can be used to better plan for land use alterations and potential cumulative impacts.

## Introduction

The Appalachian region of the eastern United States is an important source of fossil fuel to meet energy needs. Within the region, surface production of coal accounts for two thirds of total production, while underground mining contributes about one third of total production [1]. Regional coal resources include steam coal used in electric power generation, and (to a lesser extent) metallurgical coal used in industrial processes.

The overall future of Appalachian coal resource extraction is increasingly uncertain. There is a complex, dynamic relationship between the price of coal, the price of competing resources (in particular natural gas), and potential greenhouse gas emission reduction policies which reduce the demand for coal. Coal is subject to increased competition from natural gas as a source of energy for electricity generation, and may be equaled or surpassed by natural gas in the near future depending on oil and gas prices, greenhouse gas related policies, coal production costs, and other factors [2]. Coal production is also shifting geographically within the region, as demand for cleaner-burning, lower sulfur coal has risen due to increased environmental regulation.

Even with coal predicted to play a smaller and smaller role in America’s energy mix in the future [3], the need exists to model and spatially predict where surface coal mining is anticipated in Appalachia due to the potential environmental impacts of surface coal mining. Many studies have documented the impacts of coal mining on biodiversity [4, 5], hydrology [6–8], human health [9], and water quality [10–12]. The additive effects have been examined related to how multiple surface mines can impact streams [13, 14] and how the importance of spatial location and network position with other preexisting factors (other surface mines, deep mines, and residential development) can contribute to ecological stress [15–17]. By better predicting probable areas for surface coal extraction, the potential environmental impacts on sensitive ecosystems can be identified and context dependent conservation priorities can be set in complex river systems [18].

This study provides a method for predicting future surface coal mining extents by integrating a spatial model with production demand forecasts to better represent land cover change. By combining these components, a more holistic prediction can be made. This has only been recently possible due to efforts that quantified the areal extent of surface coal mining activities to coal production [19]. This enabled us to combine varying estimates of surface coal mine production [2] with spatially explicit predictive modeling to map potential future surface mining footprints on the landscape through the future. We demonstrate how the extent of surface mining can be predicted with this approach and compare the results to actual recently submitted permits.

To our knowledge, the most closely related effort related to our work was by Watson [20] who mapped remaining coal reserves with high market potential in the Pittsburgh coal bed. Our approach advances the effort in three distinct ways. The first is that the scale of our study is wider in scope than a single coal seam. We included the entire Appalachian region which covers northern, central, southern, and the eastern Illinois sub basins. Because of this we were able to create a forecast of future coal mining with a specific focus on surface mining throughout the Appalachian area. We also focused on surface mining rather than various underground techniques (such as longwall underground, room and pillar). For the past 10–15 years, surface mining has been a more common practice across Appalachia due to expansion of mountain top removal techniques [21]. Throughout the region the remaining coal seams are often too thin or deep to underground mine and the unconsolidated overlaying rock makes the roof weak for underground mining to occur safely [21]. Second, our approach incorporates forecasted demand scenarios into the model prediction which were linked to a surface area extraction ratio by region. These two developments enabled us to spatially allocate the demand across the region under different scenarios. And third, while we would have benefitted from isopachs of coal bed thickness for all the coal seams throughout our region, many of the physical property datasets we created as model variables were locally sampled values which we interpolated with geostatistical modeling to create regional datasets. This enabled us to use locally sampled input data to create interpolated datasets to address the main research question of where future surface coal mining was likely to occur throughout the regional extent.

## Methods

**Fig 1:**
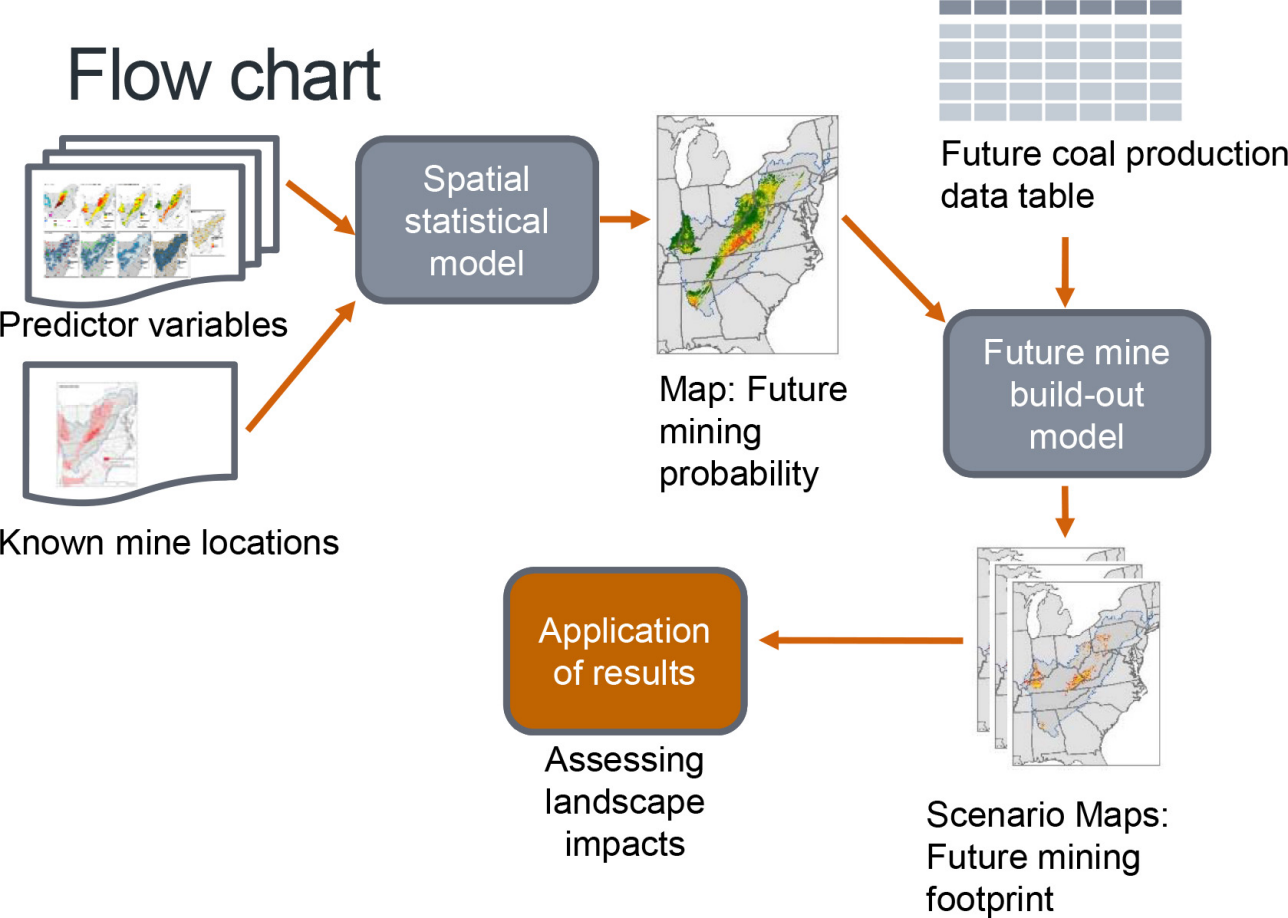
Project methodology flow chart.

An overview of the methodology which includes the spatial data collection and input datasets for the predictive model of this study are provided in Fig 1.

The methodology for this study included defining appropriate predictor variables, running a Random Forests [22] spatial model and performing predictive mapping by allocating production forecasts for a future surfaced mine footprint. A fundamental first step to this effort was to select those landscape predictor variables which can be used to effectively model the locations of future surface coal mining.

### Predictor variables

**Fig 2:**
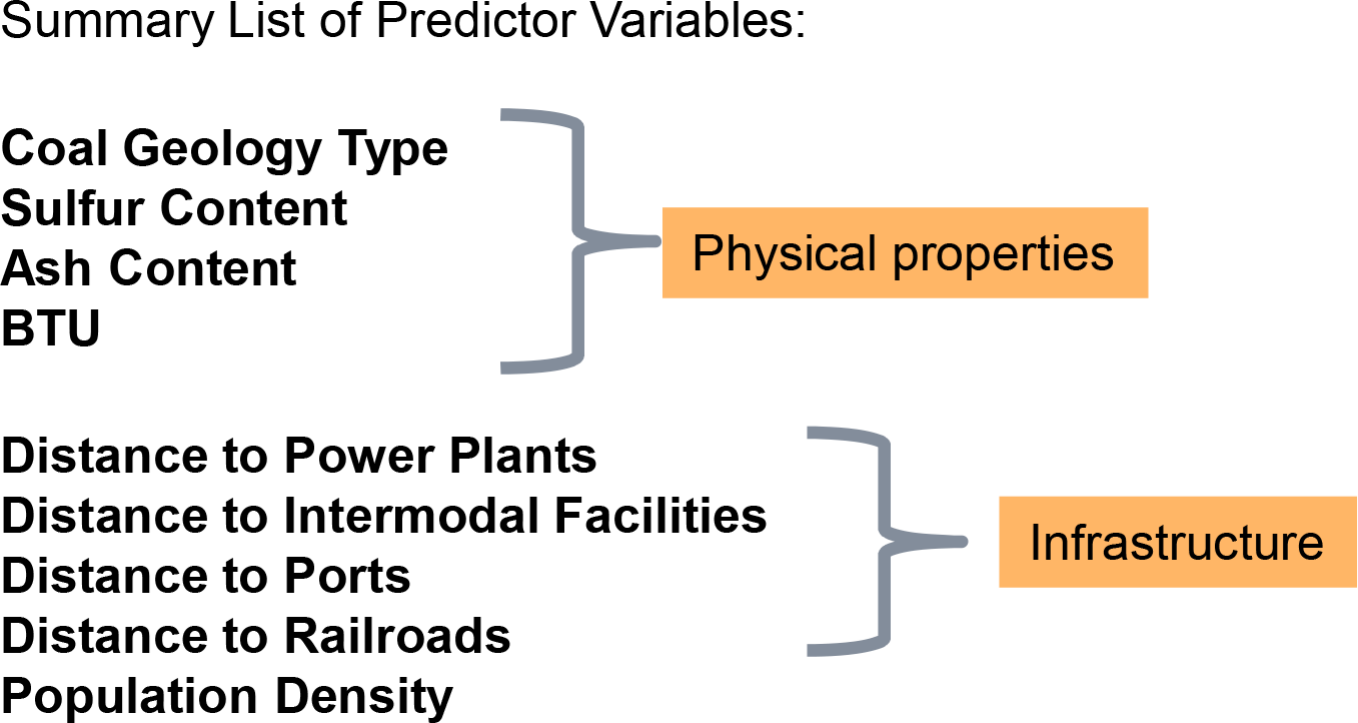
Summary list of predictor variables.

Variables included physical properties of the coal resource (coal geology type, sulfur content, ash content, and BTUs), and infrastructure related predictors (network distance to existing coal fired power plants, network distance to intermodal transportation facilities, network distance to inland ports, distance to rail, human population density). All variables were represented as raster data models with a cell size of 1 km^2^ using ESRI ArcGIS 10.1 software [23], with analysis extent limited to the coal geology extent within the Appalachian Landscape Conservation Cooperative (LCC) [24]. For distance rasters (distance to power plants, distance to railroads etc.) distances were calculated to features outside the Appalachian LCC prior to limiting rasters to the study area boundary. A summary list of predictor variables is shown in Fig 2.

#### Coal geology type

Generalized coal field boundaries were derived from a map of coal fields of the United States at a 1:5,000,000 scale [25]. Generalized coal fields include areas with known coal-bearing geology, and were used to limit the extent of predicted future mining probability within the study area (future mining was limited to areas within mapped coal fields).

Within this coal field boundary, we also obtained state level geologic maps from datasets compiled by USGS for U.S. states [26]. The generalized state level geologic maps were classified into geologic units containing coal, and those without coal. Finally, the geologic units containing coal were further cross-referenced into 17 different geological units region wide based on generalized lithology and formation. The cross referencing process was necessary due to inconsistencies and labeling among the different states. This was completed using a chronostratigraphic correlation chart [27]. Formations were grouped based on geologic age to produce 17 final mapped categories of similar lithology that are not impacted by state boundaries.

#### Sulfur percentage of coal

The sulfur content of coal is one aspect of coal quality which was important to characterize as a model variable. Restrictions on sulfur dioxide emissions from power plants have made the relative sulfur content of coal an important consideration in the economic viability of different coal resources (with low sulfur coal generally being more desirable). The percentage of sulfur content in the coal was interpolated using borehole data from the USGS Coal Quality database [28]. Prior to interpolation, borehole data were limited to samples taken at the surface (underground or deep mine samples were excluded). Underground and borehole samples (excluded) were identified by sample depth values and/or descriptive text in the comments field in the sample database. Surface samples were also identified by values in the comments field indicating samples were taken at road cuts, pits, and strip mines [28]. While different coal seams may be encountered with each of the borehole sites, an overall sulfur percentage is assumed for each site.

The interpolation process for sulfur, ash, and British Thermal Unit (BTU) followed standard geostatistical kriging steps [29]. They included first exploring the data for normality, examining trends and the semivariogram, and testing model output runs until a satisfactory root mean squared error and mean standardized error from the cross validation prediction errors were found.

For sulfur, an ordinary kriging model was applied and anisotropy examined to account for directional influences. This was useful especially since the coal geology follows ridge and topographical features. A total of ten lags were applied with a size of 20,000 to best fit the distribution of the input point locations. The search neighborhood was standard sized with a maximum of 5 neighbors. The results for sulfur cross validation indicated an accurate predicted surface with a root-mean-square standardized prediction error of 1.009 (a value closer to 1.0 is preferred [29]).

#### Ash content of coal (ash yield)

Ash content of coal is also related to relative coal quality. Ash content is related to the portion of coal that remains after combustion. Ash yield was also obtained from the USGS Coal Quality database [28] and was also interpolated using methods similar to those used for sulfur content.

For ash, again ordinary kriging was applied with anisotropy examined for the directional influences which indicated an improved fit with an angle of 44.6 and 45 tolerance. The lags used were different for ash – 12 total with a lag size of 12,000. The search neighborhood was standard sized with a maximum of 5 neighbors as with sulfur. The results for sulfur cross validation indicated an accurate predicted surface with a root-mean-square standardized prediction error of 1.001.

#### BTU content

BTU content of coal is related to the amount of energy provided by a given amount of coal. BTU content of coal per lb. was derived from the USGS Coal Quality database [28] using methods similar to ash and sulfur content.

For the BTU interpolation, a simple kriging model was applied with a log score transformation to make the variances more constant throughout the study area and bring the data closer to being normally distributed. Anisotropy was applied to account for direction in the semivariogram and covariance. The preferred angle was 32 with a 21.4 degree tolerance. Twelve lags with a size of 15,000 was found to fit the model best with the averaged data points. Again here, the standard neighborhood search was used with a maximum of 5 neighbors. The fit for BTU was not as well as ash and sulfur with a root-mean-square standardized error of 0.887.

#### Distance to coal fired power plants

Existing coal fired power plants were identified using information published by the U.S. Energy Information Administration, based on form EIA-860 Annual Electric Generator Report [30]. The locations were determined using latitude/longitude coordinates provided by SourceWatch [31] and shapefiles provided by Energy Information Administration [32]. We identified a total of 318 existing power plants as of 2011. We then removed a total of 92 of these plants that are scheduled for closure between 2013 and 2020 [31]. An additional 25 new coal fired facilities (including power plants, cogeneration facilities, coal to liquids plants) were added to the final dataset that are proposed, planned, in permitting, or under construction for this area as noted by Source Watch [31], the Sierra Club [33], and National Energy Technology Laboratory [34]. For our final predictor variable, we calculated distance along a highway network [35] to 251 coal fired power plant facilities (226 existing, 25 new). Distance along the highway network was initially calculated along 1 km^2^ cells along the actual highways, and was then extrapolated out to cover all cells within the Appalachian LCC using an inverse distance weighted interpolator.

#### Distance to intermodal transportation facilities

Intermodal transportation facilities are locations where freight may be transferred between different modes of transportation (i.e. truck to barge, truck to rail, etc.). Intermodal facility point locations were obtained from the National Transportation Atlas Database, and were then limited to all facilities except ports, which were mapped separately [36]. Distance to intermodal facilities was mapped along the highway network, then extrapolated out to all cells within the Appalachian LCC.

#### Distance to inland ports

Inland river ports were also obtained from the National Transportation Atlas Database [36] and were limited to those ports handling coal and coal related commodities. Distance to ports was mapped along the highway network, then extrapolated out to all cells within the Appalachian LCC.

#### Distance to rail

According to U.S. Energy Information Administration domestic coal distribution statistics, 56% of coal produced by the ten coal-producing states in the study area was distributed using rail in 2011 [32]. In addition, a total of 29% of coal distributed domestically was moved by river (barges), with a total of 13% was transported by truck. This implies that proximity to rail, river, and trucking related loading facilities may be an asset in location of potential mining activity. Mining related facilities (for loading coal onto rail cars) are not necessarily limited to locations at end points of rail lines. Mine loading facilities can also be found at any point along rail lines, not just at the end points or at spurs. Mapping distance to existing rails captures more potential locations for access to rail lines from coal mining permit locations, rather than limiting the rail feature dataset to endpoints only of existing railroads.

Locations of railroads were acquired from the Bureau of Transportation Statistics U.S. National Transportation Atlas railroads layer, at the 1:100,000 map scale [37]. Distance to nearest rail line was mapped as Euclidean straight line distance across the Appalachian LCC (not limited to distance along network).

#### Population density

Population density was calculated across the study area using 2010 Census block group data, and was then converted to raster format, 1 km^2^ cell size [23].

#### Other data considerations

Economic factors are important to consider for future mining since development decisions have costs ultimately built into the decision process. Most of the economic variables for mining are related to the deposit geometry stripping ratio, size, shape, and depth of strike of deposit, rock conditions, productivities and machinery capacities as well as some of the more common economic costs related to capital requirements and operating costs, discount rate, investments, amortization, depreciation, recoveries and revenues, labor force availability, and environmental regulations [38]. Other factors considered to be important for new surface mining activity included past and existing mining, stripping ratios (overburden, coal bed thickness), coal reserves remaining, surface ownership patterns, and coal quality as related to market demand. Each of these factors were specifically mentioned by internal reviewers in various stages of this project, and were also mentioned in the Environmental Impact Statement for mountaintop removal mining in the Appalachian region [39]. Ultimately, these factors were not included (directly) in the final modeling process, after investigation of available datasets and data quality. Location and extent of past mining were not uniformly available for the entire study area, as mining datasets from individual states varied greatly in quality. Data related to stripping ratios (overburden, seam thickness) were available for some coal seams [40] and states in the study region (Illinois [41]; Indiana [42]; West Virginia [43]; Virginia [44] but not others. Remaining coal reserves are available on a county-by-county basis for some states (see [45] for example) or on a regional level from the U.S. Energy Information Administration, but reserve data are not consistently published at a detailed enough spatial scale for the region in order to be included in the project. The focus of our modeling on surface mining activity only (rather than surface and underground combined) also placed more importance on overburden coal amounts as well as accessibility from the surface.

For surface land ownership patterns, it has been suggested that the differing nature of land ownership among states may be related to surface mining–specifically that surface mines of eastern Kentucky are characterized by smaller land owners, while surface mines in neighboring southwestern West Virginia are more likely to be owned by larger corporate land owners [39]. Based on a quick cross reference with existing permit data, we did not find this to exist as the average permit size in Kentucky was larger than the average permit size for West Virginia. In any case, land ownership data for such a large study region is nearly impossible to assemble, particularly in light of the relatively coarse spatial scale of this work (1 km^2^ cell size). We also did not have access to adequate mineral rights data for the entire study region, another important consideration. While these data limitations preclude our ability to make the same local decisions a coal company would make for a site, the goal of this project was to focus on broader regional predictions and forecasting.

### Active surface mine permit locations

The previously listed independent variables were analyzed with the dependent variable of location of active surface mine permits. The centroids of each permit were calculated for the model runs. Surface mining permit locations were obtained from individual state agencies for the ten coal-producing states within the study area. Mining permits were further limited to active surface mining permits only by excluding underground mines and permits associated with inactive or historical mines. In certain states, if permit status (active/inactive) was not indicated, permits were limited to those with dates from the year 2000 to the present only, in an attempt to limit analysis to current, active mines.

### Exclusion areas

In addition to the above mentioned predictor variables and surface mine permit locations we also integrated spatial data sets as “exclusions” or areas where surface coal mining could not occur. Areas excluded from our predictive modeling of future surface mining include permanent conservation lands and areas with existing land uses that are not conducive to mining activities (urban and developed lands, water) based on the 2006 National Land Cover Dataset [46]. For purposes of this work, we considered permanent conservation lands to be (in most cases) lands compiled in the Conservation Biology Institute’s Protected Areas Database [47] with Gap Analysis status 1 or 2. Conservation lands with Gap Analysis status 1 and 2 [48] generally indicate areas with permanent protection from land use conversion and/or management plans designed to limit disturbance and may include national parks, national wildlife refuges, state parks and preserves, and U.S. Forest Service wilderness areas (among others), although further assessment of outstanding mineral leases on these tracts may result in their re-inclusion in the area where mining may occur. In all, 57,185 km^2^ throughout the study area (9.6%) was excluded due to land use restrictions, while 14,366 km^2^ of the study area was excluded due to presence of conservation lands (2.4%).

Areas were also identified that contained an extensive recent history of surface mining, as we are assuming these areas to be “mined out”, meaning they will not be surface mined again in the future. Mined out areas were identified as cells within current active surface mine permits that were classified as Barren land cover in the 2006 National Land Cover Dataset [46]. This method ensured we were capturing large contiguous areas of previous surface mining, and not newly opened mines (since we were using 2006 land cover). By using this method, we excluded mining on a total of 567 km^2^, or 12% of the area contained within active surface mine permits.

### Predictive coal model

We used the non-parametric model, Random Forests [22], to estimate surface coal development probability, for each of the 1 km^2^ cells, with higher probabilities indicating a greater likelihood of future mining. The Random Forests algorithm offers many advantages in that it does not adhere to parametric assumptions, can utilize mixed data type with different scale, handles high dimensional data, is robust to outliers and noise, is not sensitive to autocorrelation, quantifies importance of the predictor variables and requires minimal parametrization [49–52].

The Random Forests model is a weak-learner ensemble approach, where a series of unconstrained Classification and Regression Trees (CART) are created using a bootstrap sample with replacement. The CART’s are constructed using an entropy node splitting statistic that recursively partitions the data into more homogeneous subsets and results in a hierarchical classification that accounts for 1st and 2nd order statistical variation. The out-of-bag (OOB) data withheld in each bootstrap sample is used to assess fit, at each model iteration, and results in convergent fit statistics without requiring data be withheld for validation. The independent variables are randomly permutated through the nodes and mean decreases in accuracy thus accumulate providing an importance measure of each variable. The plurality of votes across the ensemble converges on the optimal fit to the data and provides a robust estimate [49].

To specify a binominal response variable (y) where; presence (surface mining has occurred during the permit period) and absence (no surface mining has occurred during the permit period), we utilized surface mine permit centroids, only using observations permitted after the year 2000. To ensure that statistical and spatial variability was represented without introducing a zero-inflation issue [49], we created five sets of pseudo-absence data by creating random points and then removing observations occurring within a current-permit or 0.5 miles of a surface mine centroid. For each training subset, we used an equal number of presence (n = 5,165) and absence (n = 5,165) observations, with the same presence data used in each subset. The independent variables were appended to the points, from the corresponding raster cell(s), using the software tool Geospatial Modeling Environment [51].

Using the complied training data we specified five Random Forests models, representing each random subset, using the Random Forests [53] package in R [54]. We tested models by removing low-performing parameters and observed a decrease in model performance as compared to the full model. Model error converged in fewer than 1,000 bootstrap replicates however, since variable interactions stabilize at a slower rate than error, we fixed the number of bootstrap replicated at n = 1,000. Because Random Forests is an ensemble approach, as long as the parameter space remains fixed, independent models can be combined into a single ensemble-model [52]. Using only consistently selected parameters in the model selection, we fit final models for each random-subset and combined them into a final ensemble-model. Model significance was evaluated using a permutated (n = 999) randomization procedure and an iterative 10% withhold cross-validation using the rfUtilties R package [55]. The probability of the presence class {1} was predicted, using the scaled posterior distribution of the vote plurality [50], with the R raster package [54]. The estimated extent was limited to the known extent of coal in the region.

### Predictive Mapping: Future Surface Mining Footprint

In order to map future potential surface mining activities on a landscape scale, we used results from the probabilistic Random Forests modeling of surface mine potential along with regional-level estimates of future coal mining production for the years 2012 through 2035.

**Fig 3:**
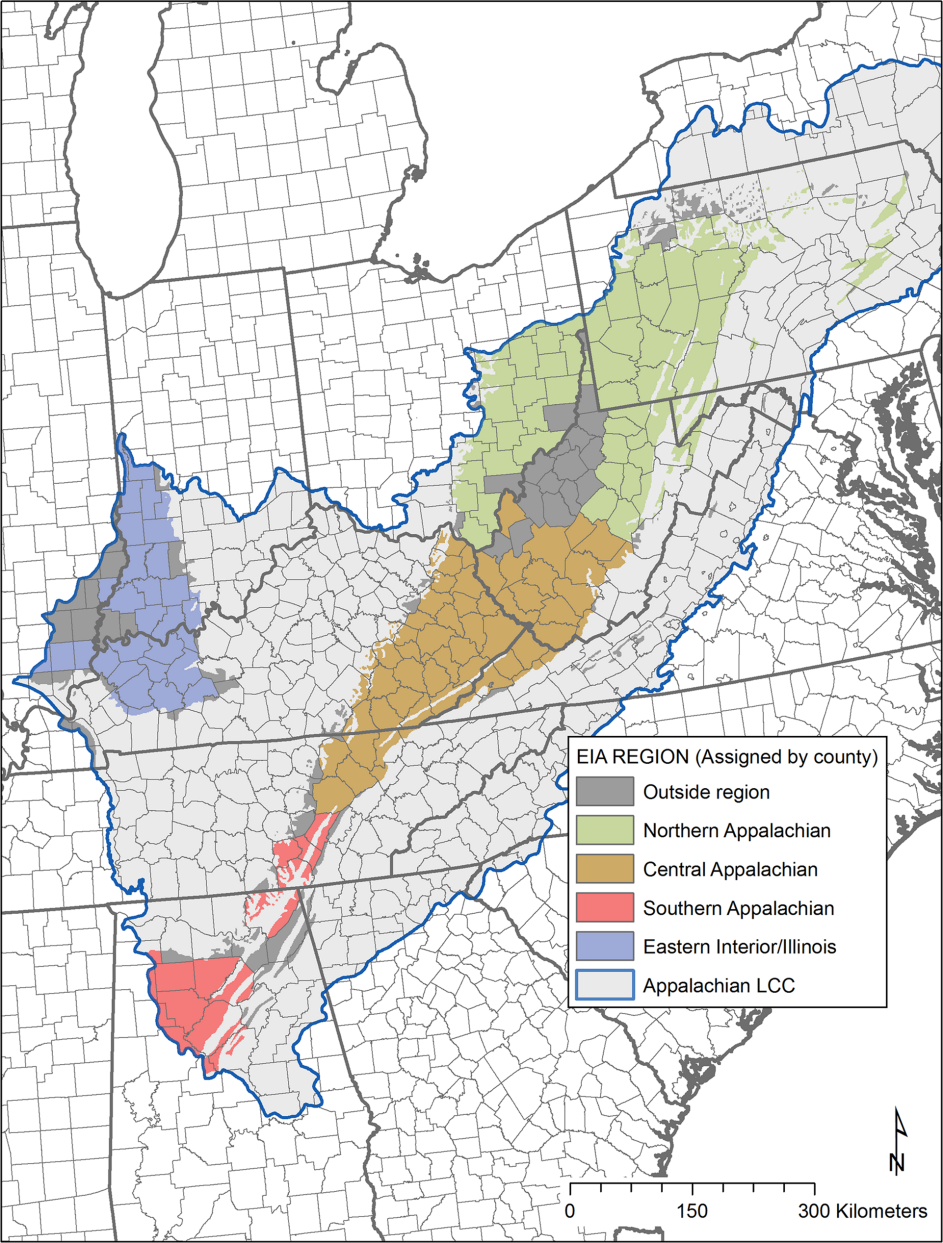
U.S. Energy Information Administration (EIA) coal supply regions within the Appalachian Landscape Conservation Cooperative boundary used in this project. Counties contained within EIA coal supply regions (Northern, Central and Southern Appalachian, Eastern Interior/Illinois) were determined from the EIA Annual Energy Outlook [2]. The area modeled was further limited to the intersection of the coal supply regions with generalized coal field boundaries for the United States, obtained from the U.S. Geological Survey [25]. This figure shows the intersection of the coal supply regions with actual coal field boundaries. The boundary of the Appalachian Landscape Conservation Cooperative is shown as a thin blue line, obtained from the U.S. Fish and Wildlife Service.

Regional coal production estimates for the four EIA coal supply regions (northern, central and southern Appalachians, eastern interior/Illinois) (Fig 3) were obtained using various coal production scenarios from the EIA’s Annual Energy Outlook [2]. Values were obtained for two different EIA economic/coal production scenarios for comparison: a low coal production scenario and a high coal production scenario. The low coal production scenario (“GHG25+low gas”) predicts the lowest future coal production of any of EIA’s 28 total scenarios, due to very restrictive greenhouse gas emissions policies and low prices for competing resources of oil and gas. The high coal production scenario (“low coal cost”) predicts the highest coal production due to lower costs for coal mining wages, transportation, and mine equipment (leading to increased coal production).

EIA coal production estimates provide total production estimates only (surface and underground combined). We limited future production projections to surface projections only by multiplying each production total by the percentage surface according to the following regional figures based on 2010–2011 production data in the Annual Energy Outlook: northern Appalachians: 20.08% surface, central Appalachians: 48.68% surface, southern Appalachians: 40.06% surface, eastern interior/Illinois: 30.72% surface. Surface mining production estimates from the year 2012 through the year 2035 were then summed to produce a total cumulative surface coal production value for each region.

In order to estimate surface area impacted by coal mining activities, a numeric relationship was required between surface mine production amounts and a corresponding area disturbed. It was initially proposed to use current active surface mine permit data along with recent production statistics in order to derive a production to area ratio. However, single mines may produce coal for extended periods of time, and this method would not adequately capture the entire life cycle of a mine. In addition, mapped mine permit polygons may include areas that are not actually disturbed during surface mining, so the actual disturbed area may be much smaller than mapped permit area. A recent study concluded that mapped mine permits do not offer an accurate way to estimate area disturbed by surface mining, based on current permit database and mapping methods used in WV and KY [56]. Instead, Lutz et al. [19] developed a regression model to estimate tons of coal produced per unit areal disturbance for 47 counties in southern WV and eastern KY. The model was based on total area of surface mining disturbance from 1985–2005 (at 5 year time intervals), compared with surface coal production statistics for corresponding time periods. Lutz et al. [19] estimated that 1 ton of coal equates to 0.87m^2^ of surface disturbance. For the current study, this figure was converted to 1.15 million tons of coal produced per square kilometer of surface land disturbance.

Future surface mining scenarios analyzed included low coal production and high coal production models [2] for the years 2012–2035. For each scenario, we created a new map layer showing potential locations for future surface mining activities on a cell-by-cell basis using a 1 km^2^ grid for the study area. Using the figure of 1,150,000 short tons per km^2^, we allocated future mining production on a cell-by-cell basis within each EIA region first to those cells with the highest future mining probability, then continuing to cells with lower future mining probability, until the total amount of future production for a particular scenario and region was allocated. Prior to allocation, adjacent cells with identical mining probability values were grouped together to ensure that contiguous areas of high mining probability were preserved in the results (rather than assigning “new” mining to single cells). Cells containing urban or built up land, water, conservation lands, and centroids of existing mining permits were excluded (masked out) prior to build-out analysis as described earlier.

## Results

### Random Forests Model (Probability of Future Surface Coal Mining)

The final Random Forests model scenario included the original 9 predictor variables of Fig 2. We experimented by removing low-performing variables from the model based on variable contribution to the overall result. However, alternative models with fewer variables did not perform as well as the full model, producing higher classification error rates. Model significance was tested vs. randomly generated models and was found to be significant p = 0.01.

**Fig 4:**
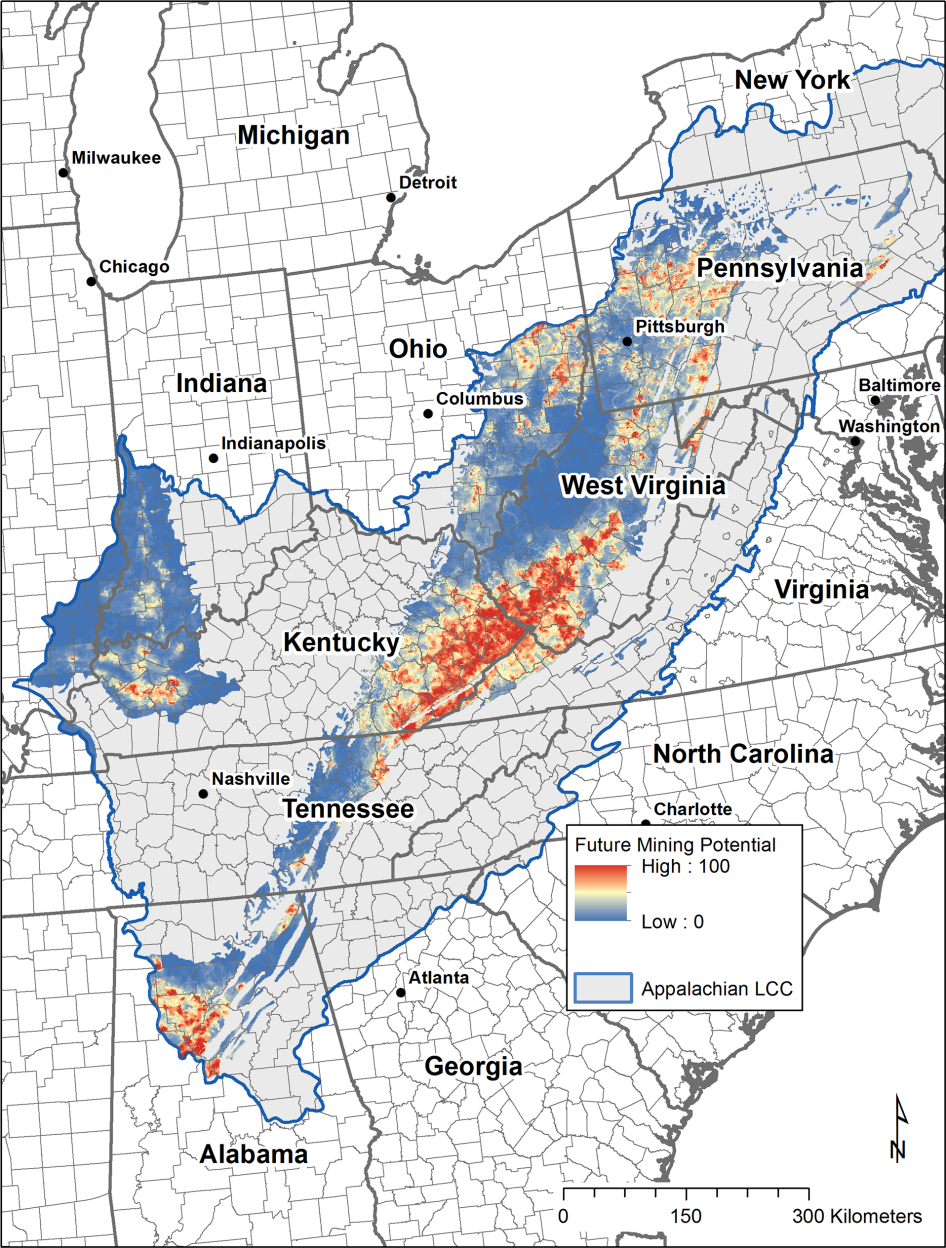
Surface mining probability from Random Forests model results. Model extent was limited to the known extent of coal in the region (coal field extents obtained from U.S. Geological Survey) [25]. Random Forests model result ranges from 0 (lowest modeled probability of future surface mining activity) to 100 (highest probability), shown here in a blue to red color ramp.

**Fig 5:**
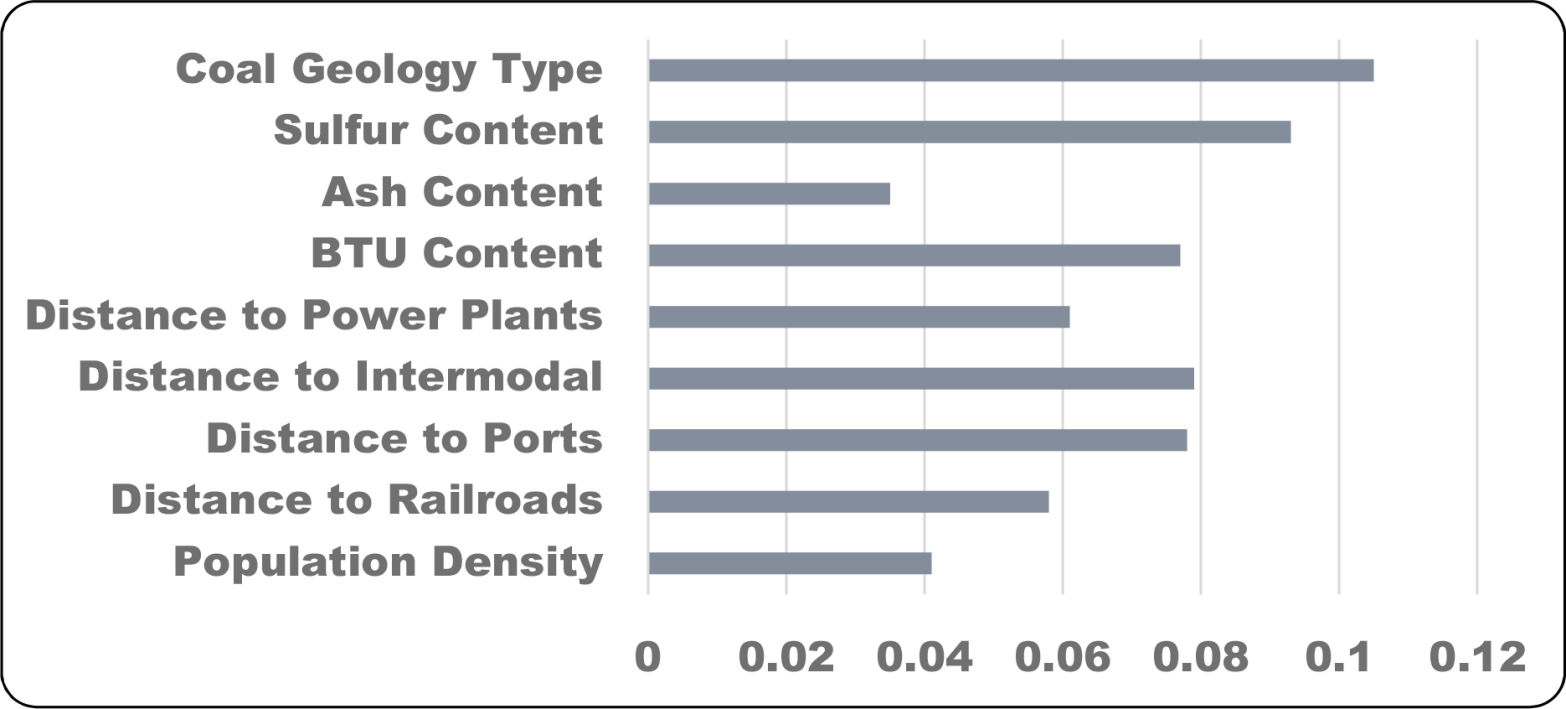
Importance of predictor variables measured as out-of-bag mean decrease in accuracy. Based on Random Forests model results.

The final output of the Random Forests model is a pixel based probability of future surface mining presence (Fig 4). As estimated by the out-of-bag mean decrease in accuracy, the coal geology type and the sulfur content were found to be the most important predictor variables in the model, though all variables contributed (Fig 5). For each training dataset, the out-of-bag error estimate was around 15% and the misclassification of presence and absence points were evenly balanced. Plotting the error rate against the number of trees generated suggests that 1,000 trees per set is more than ample to stabilize the result.

Results indicate that the highest probability of future surface mining is found in the central Appalachian region, particularly throughout southwestern West Virginia and eastern Kentucky. Other locations of higher probability are found in western Kentucky, central Alabama, and to a lesser extent, north central West Virginia and the bituminous coal region of Pennsylvania and Ohio.

The total area within each EIA coal supply region with relatively high probability (over 0.90 as defined in this study) is listed in Table 1. The central Appalachian region has the most high probability areas for the four regions, while the northern Appalachian and eastern interior/Illinois regions have a very small amount of their area within high probability. Note that while the northern, central and southern Appalachian regions lie completely within the current study boundary (Appalachian LCC), the eastern interior / Illinois coal supply region also includes production in portions of western and central Illinois and Mississippi that are not included in the Appalachian LCC study area for this project. Based on the most recent available coal production statistics from 2011 [57], there are a total of six counties in the eastern interior/Illinois region that produce coal but are located outside of the project study area. For 2011, these six counties accounted for 11.7% of the total surface coal production for the eastern interior/Illinois region (so approximately 11–12% of coal production in this region will not be accounted for in our model results and projections).

**Table 1:** Supply regions and probability (prob) results.

Region Name	Area (km^2^)	Area < 0.90 prob	Area > = 0.90 prob	Percent < 0.90 prob	Percent > = 0.90 prob
**northern Appalachian**	68,852	68,385	467	99.32	0.68
**central Appalachian**	53,788	49,368	4,420	91.78	8.22
**southern Appalachian**	14,455	13,635	820	94.33	5.67
**eastern interior/Illinois**	28,147	28,048	99	99.65	0.35

EIA coal supply regions, with area of relatively high (0.90 or higher) probability of future surface coal mining, based on Random Forests model results.

### Predictive Mapping Future Surface Mining Footprint

**Fig 6:**
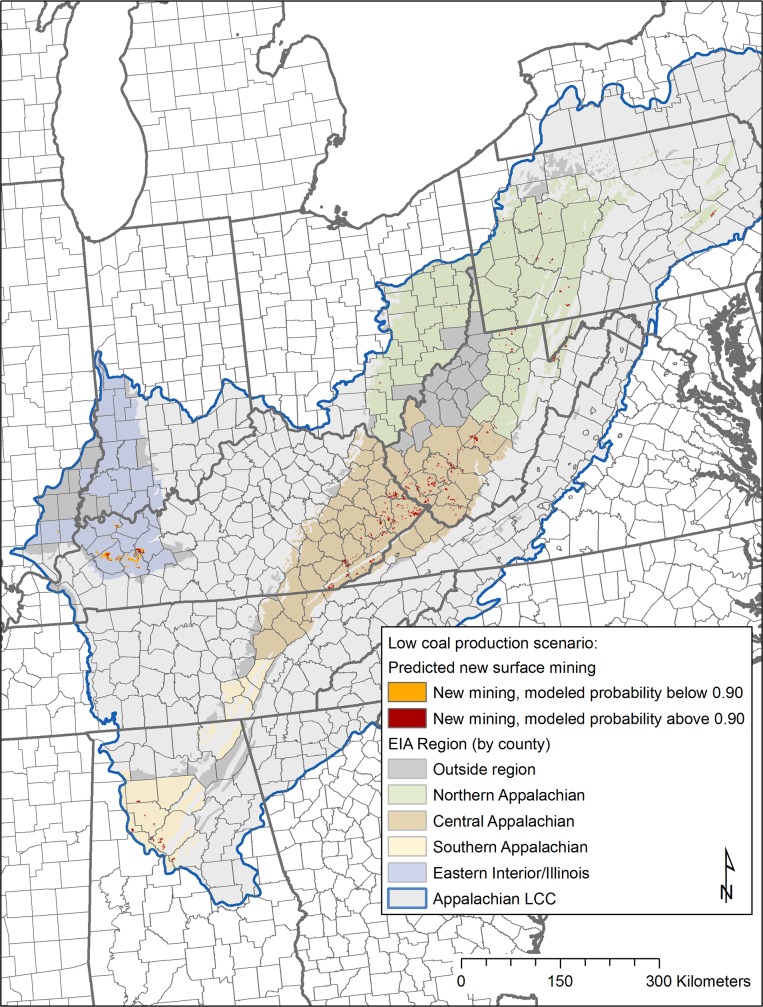
Low coal production scenario. Future surface mining spatial footprint in order to meet coal production estimates for low coal production scenario through 2035 (based on EIA GHG25+low gas price scenario) [2]. Areas with predicted new surface mining through the year 2035 with modeled probability < 0.9 (based on Random Forests results) are shown in orange, areas with modeled probability > 0.9 are shown in dark red. Total area (km^2^) required to support new surface coal production were determined from an area to production ratio of 1.15 million short tons of coal production per km^2^ disturbed based on Lutz et al. [19].

**Fig 7:**
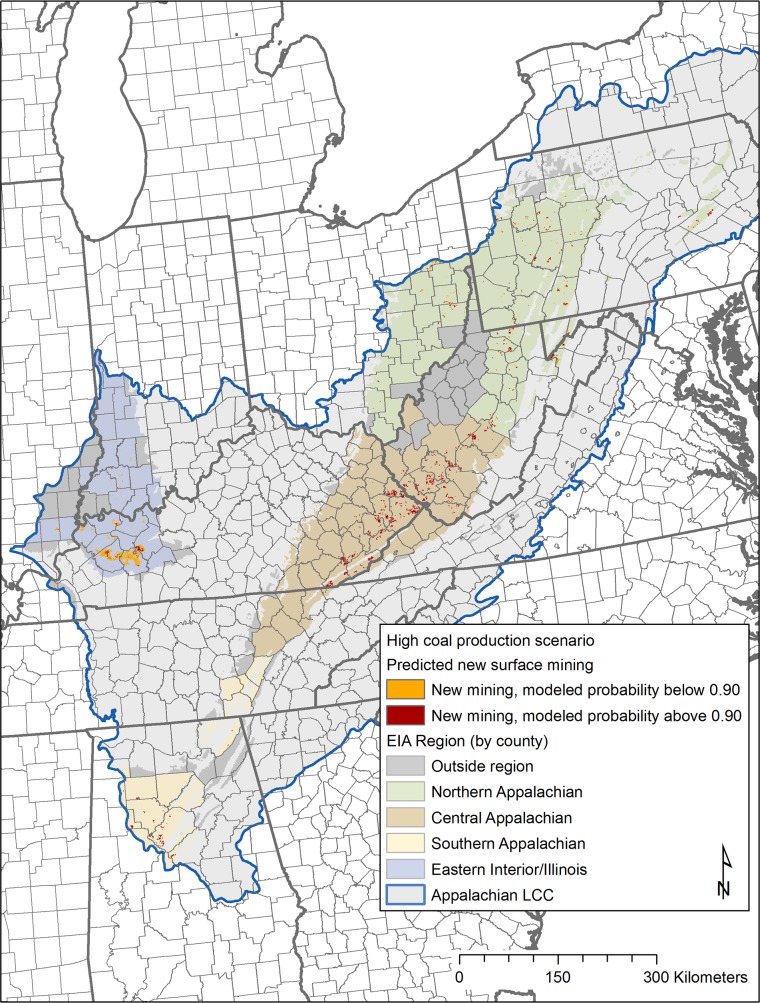
High coal production scenario. Future mining footprint for coal production through 2035 (based on EIA low coal production cost scenario or high coal production). Future surface mining spatial footprint in order to meet coal production estimates for high coal production scenario through 2035 [2]. Areas with predicted new surface mining through the year 2035 with modeled probability < 0.9 (based on Random Forests results) are shown in orange, areas with modeled probability > 0.9 are shown in dark red.

Results for future surface mining footprint by the year 2035 are shown in Fig 6 (low coal production model: GHG25+low gas) and Fig 7 (high coal production model: low coal cost). Total area (km^2^) mapped as new surface mining activity is listed by EIA region in Table 2. We also determined the high probability area affected by new mining for each region. For the low coal production scenario, all regions except the eastern interior/Illinois are predicted to have all new mining footprints located completely within our defined high probability areas. For the high coal production scenario, only the central and southern Appalachian regions are predicted to have all new mining footprints found within higher probability areas.

**Table 2:** Production scenarios by region.

	Low coal production scenario		High coal production scenario	
Region name	Mapped new mining (km^2^)	Probability level needed to allocate all new mining in the region	Mapped new mining (km^2^)	Probability level needed to allocate all new mining in the region
**northern Appalachian**	272	0.91	776	0.85
**central Appalachian**	953	0.97	1,186	0.96
**southern Appalachian**	103	0.99	148	0.99
**eastern interior/Illinois**	453	0.68	991	0.54

By EIA coal supply region, total area mapped as new surface mining under differing scenarios (low coal production, high coal production). Minimum Random Forests probability result needed to allocate all new mining is given by region

**Fig 8:**
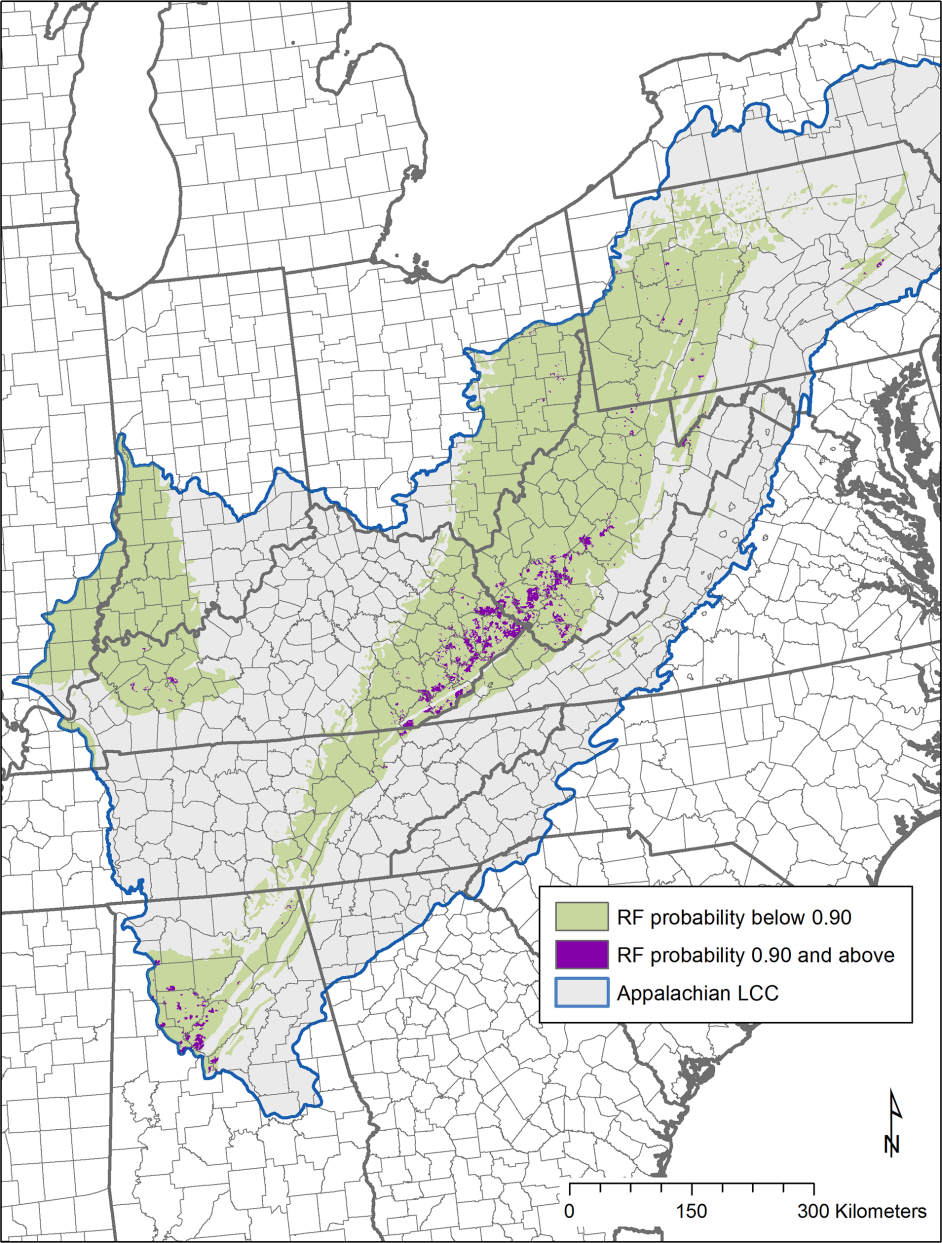
Random forests model result. Dark purple areas indicate results of Random Forests modeling as areas with high likelihood of future surface mining (probability 0.90 and above) with mapped coal fields (green).

To meet production estimates for the low coal production scenario, the three Appalachian regions are each predicted to have all new surface mining development limited to high probability modeled areas. These highest probability areas (shown in Fig 8) are concentrated in southwestern West Virginia and eastern Kentucky, with a significant portion in Alabama (southern Appalachian region). However, surface mine footprints within the eastern interior/Illinois region may need to extend beyond the highest modeled probability areas in order to meet projected production figures (according to model results, the area required to meet future coal production in this region has a minimum probability score of 0.68 (Table 2). Within this region, under the low coal production scenario, new mining is modeled to occur in lower probability areas concentrated within Hopkins, Henderson, Ohio, and Muhlenberg counties in western Kentucky.

In order to meet the future high coal production scenario, the area associated with future coal production for both the eastern interior/Illinois and the northern Appalachian regions exceeds the current high probability area for those regions. In the northern Appalachian region, in order to meet high coal production predictions, new mining is modeled to extend into areas with a minimum model probability of 0.85 (Table 2). These areas are found scattered across counties in eastern Ohio, western Pennsylvania, and north central West Virginia. In the eastern interior/Illinois region, new mining is modeled to extend into areas with a minimum model probability of 0.54 (Table 2). Within this region, new mining areas are again concentrated in western Kentucky, with smaller amounts in Illinois (similar to the low coal production scenario).

## Discussion

In an effort to compare our model results for locations of future surface coal mining activity with established data, we compare our results with three related sources of data: coal seam level data (coal availability/thickness), remaining coal reserves, and newly permitted areas. These datasets were not used in the model creation because they were not uniformly available across the entire study area.

### Coal seam level data

Data on individual coal seams are available from multiple state geological survey agencies as well as the U.S. Geological Survey. Mapped coal seam properties include coal seam depth to top of the seam, seam thickness, and overall coal availability. Mapped properties vary by seam.

**Fig 9:**
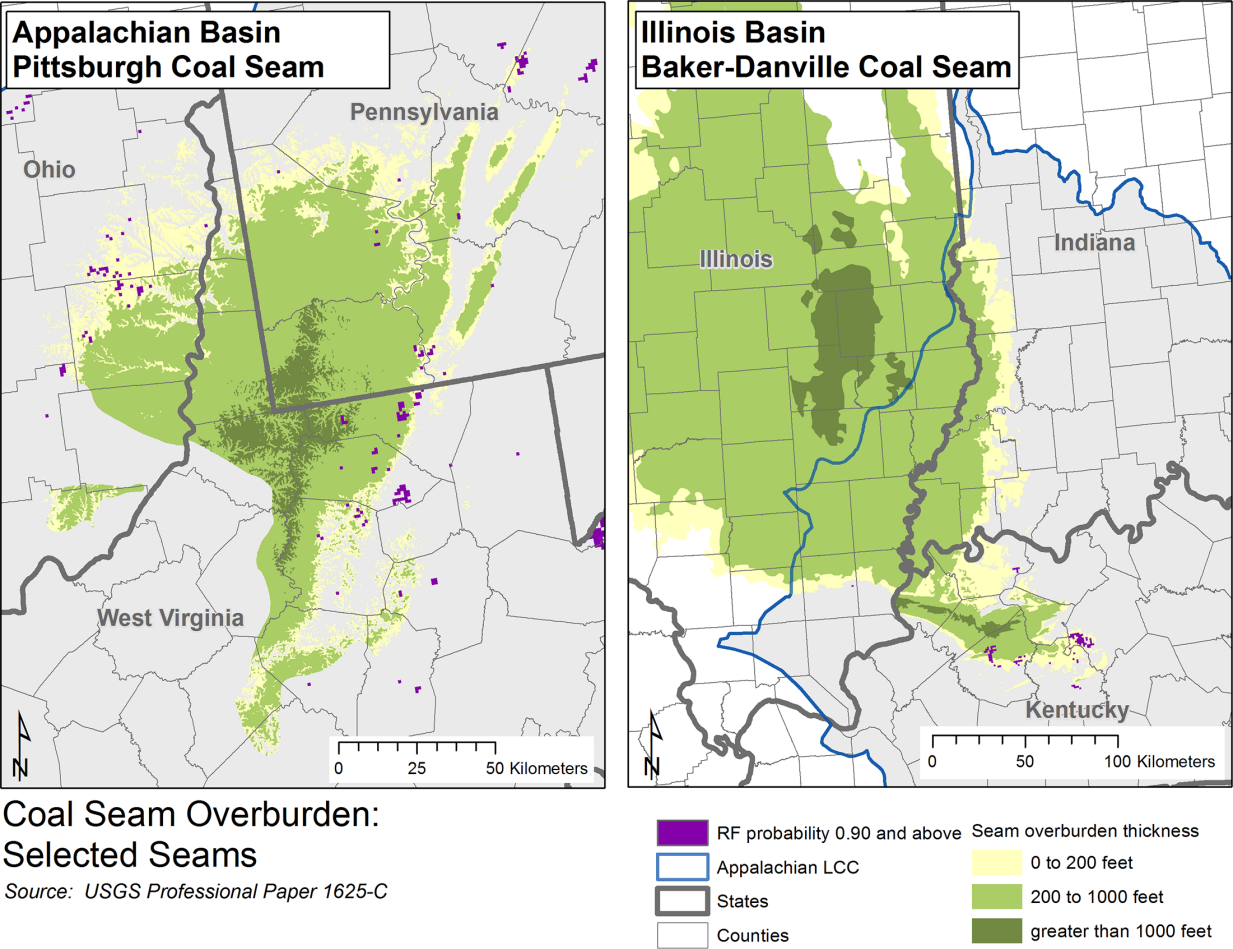
Overburden of coal seam comparison to random forests model results. In general, coal seams with higher amounts of overburden indicate increased costs for mining and recovery of coal resources. Coal seam overburden data were obtained from U.S. Geological Survey [40] for selected seams including the Pittsburgh coal seam (within the Appalachian basin) and the Baker-Danville coal seam (within the Illinois basin), shown here. Modeled areas of high probably of future surface coal mining (Random Forests probability > = 0.90) are shown for comparison, and are indicated by dark purple.

USGS data on overburden and seam thickness are available for six major producing seams in the Appalachian Region [40] and three major seams of the Illinois coal regions [58]. Of these seams, three Appalachian seams (Pittsburgh, Upper Freeport, and Fire Clay) have some areas with less than 61 meters of overburden which may theoretically be available for future surface mining. A visual comparison of the level of overburden of these three seams with modeled potential areas for future surface mining (model results) showed the model tended to predict that future surface mining will be concentrated in areas of lower overburden, particularly for the Pittsburgh seam (Fig 9). Similar results were found for the three mapped seams in the Illinois region (Baker-Danville, Herrin, and Springfield coals): the model predicts future surface mining to be more prevalent in areas of lower overburden, particularly in western Kentucky (Fig 9; Baker-Danville seam).

### Remaining coal reserves data

Comparison of model results with published coal reserve figures indicates close locational correspondence between areas of future high surface coal production (from this model) and established coal reserves. County-level coal reserves (amount of remaining coal) have been published for many of the states within the Appalachian LCC study area. Model results for future surface mining probability may be compared with county reserve data for West Virginia, Pennsylvania [59], Kentucky [60], and Ohio [61].

**Fig 10:**
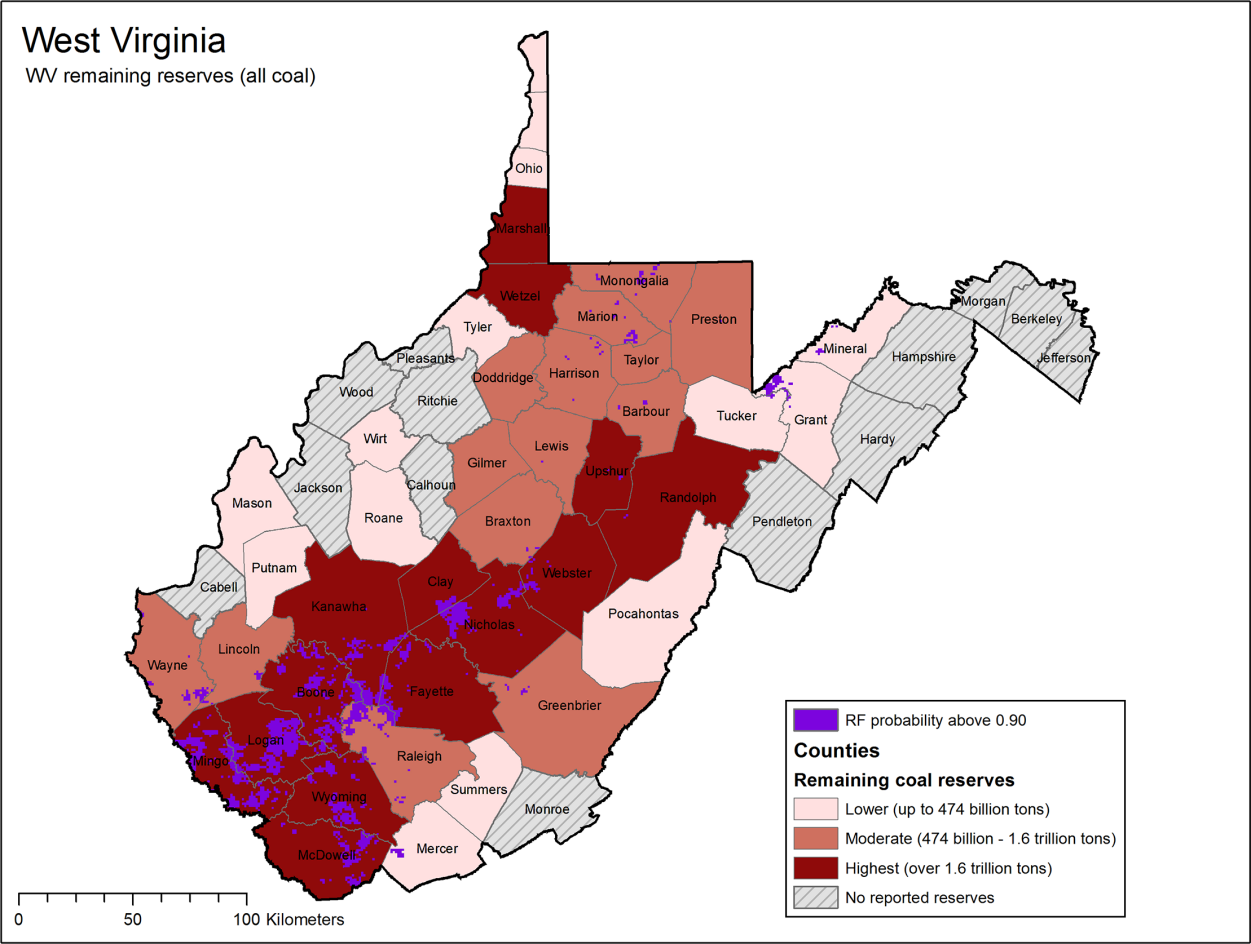
Remaining coal reserves for West Virginia counties comparison to Random Forests model results. Remaining reserves (surface and underground) for WV counties were obtained on a county basis from the WV Coal Association [45]. Modeled areas of high probably of future surface coal mining (Random Forests probability > = 0.90) are shown for comparison, and are indicated by dark purple.

Within West Virginia in particular, reserve data available from the WV Coal Association (for all types of coal–surface and underground) [45] indicate that areas mapped as high probability for future surface mining correspond strongly with counties with high remaining reserves in southwestern West Virginia (Fig 10). Similar patterns were observed in Kentucky, where counties with highest remaining reserves, particularly along the Kentucky/Virginia border, showed large areas of high probability of future surface mining.

### Newly permitted areas

**Fig 11:**
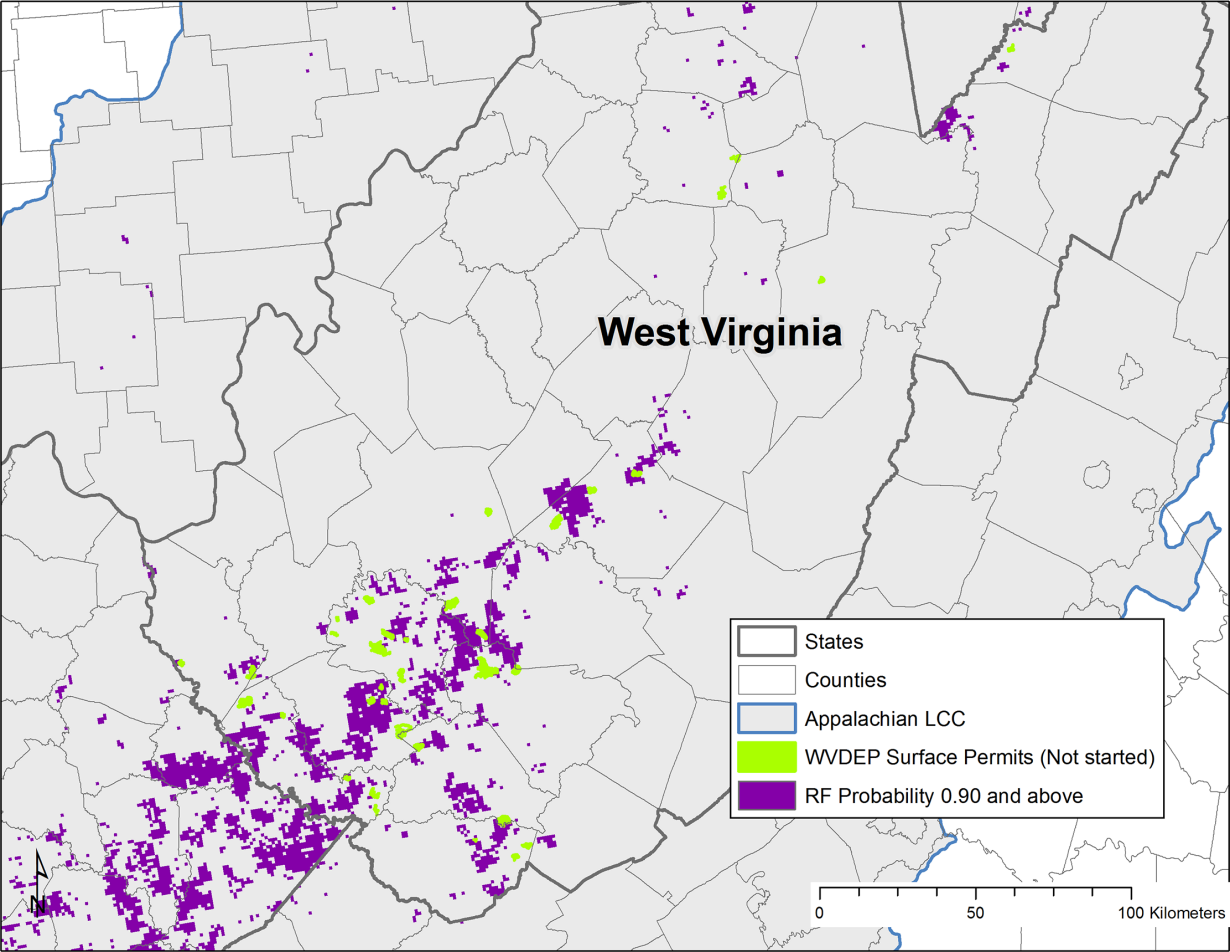
Newly permitted surface mines in West Virginia comparison to Random Forests model results. Newly permitted surface mines were obtained from the WV Department of Environmental Protection, and include only surface mine permits with status indicating the mine has been permitted, but active mining has not yet begun as of 2013. Modeled areas of high probably of future surface coal mining (Random Forests probability > = 0.90) are shown for comparison, and are indicated by dark purple.

Areas of recent surface mine permit activity may also be used to qualitatively evaluate model results. Areas modeled to have high probability of future surface mining should theoretically be associated with areas of high current permit activity (newly approved permits, permits approved but not yet started etc.). Recent permit activity was available for Alabama and West Virginia. Within Alabama, the Alabama Surface Mining Commission (ASMC) lists recent permit decisions, including renewals, revisions and applications [62]. Based on information from the ASMC, there are 86 permit polygons within the Appalachian LCC study area in Alabama that have recent permit activity in 2013 (permit activity includes renewal, revision or approval). Some permits consist of more than one polygon. Of these 86 polygons, 77 (89.5%) intersect areas of high future mining likelihood (probability over 0.90 as modeled) (Table 3). For West Virginia, recent surface permits that are mapped but have not been started yet [63] may also be used in a similar fashion. Within West Virginia, there are a total of 43 surface mine permits that have been issued but have not yet been started, and of these, 26 (60.4%) intersect areas of high future mining probability as modeled (Table 3). The vast majority of these newly permitted areas are in the southern coalfields region of the state, corresponding well with highest future mining probability areas (Fig 11).

**Table 3:** Comparison of results to new permits.

State	Permits/polygons applicable	Average RF probability for permits/polygons	Number of permits/polygons intersecting high likelihood areas (RF prob > .90)
Alabama (permit polygons with 2013 activity)	86	0.96	77
West Virginia (permits approved but not started)	42	0.80	26

For Alabama and West Virginia, detailed summary of mine permits with new/recent activity in relation to Random Forests (RF) model results.

## Conclusions

The main contribution from this work was predicting a future expansion of a critical energy extraction industry–surface coal mining in the broad Appalachian region of the United States. This was done by developing a spatial model to predict future surface coal mining extents under alternative economic and regulatory scenarios through the year 2035. The spatial model included coal production forecasts allocated across the predicted areas of high probability of future surface coal mining.

Through the modeling process, we determined that key determining factors of future mining locations at the regional scale include coal geology type, coal sulfur content, coal BTU content, and distance to transportation related infrastructure. The extent of future surface coal mining will vary regionally, with highest probability areas concentrated in the mountaintop removal/valley fill mining region of central Appalachia.

The results from this work allow for regional scale policy evaluation. From our output it is not possible to make local implications or decisions regarding an individual mine site and its probability for construction. However, this study successfully identified regional areas and opportunities to plan for possible future land conversion. Having the results for such a large regional extent as the Appalachian coal area enables broader and more encompassing analysis. This enables scientists to focus on this area at the landscape scale to evaluate how the future land cover change may impact ecological and biodiversity indicators. If projected land conversion exists in regionally important high value terrestrial habitat it may be noted as a high conservation priority and conversely if projected land conversion exists in a lower value ecological landscape it may provide an opportunity for mining companies to possibly acquire a permit to mine sooner. It may also help federal or state regulatory officials to target pre-mining reference sites for water quality evaluations.

In addition to the impacts on the natural environment from the extent of surface coal mining in Appalachia, it is important to acknowledge the future impacts that coal brings to communities in Appalachia. Recent research has examined and tested the hypothesis of a “resource curse” in which the abundant energy source of coal also brings negative effects such as reduced economic growth and associated potentially weak local government, lower education levels, impacts on human health and environmental degradation [64–69]. Using the results from this study it is possible to estimate or quantify county level changes in future surface coal mining activity. These forecasts in turn may be used to examine potential socioeconomic impacts of coal production as explored in related studies.

The analyses conducted here builds on previous energy extraction industry assessments conducted by The Nature Conservancy in the Appalachians for both wind and shale gas extraction [70, 71] which also modeled using 1 km^2^ raster cell sizes. By providing a model to estimate the future surface mine extent from this analysis, combined with the previous completed wind and shale gas models for this region, it is now possible for an informed and constructive conversation among industry, regulatory agencies, and the public regarding the importance of developing an interlocking framework of voluntary practices, comprehensive planning, and sensible regulation to ensure that extraction of the region’s highly desirable energy resources evolves in a sustainable and equitable way.
